# Cross-Species Extrapolation of Prediction Models for Cadmium Transfer from Soil to Corn Grain

**DOI:** 10.1371/journal.pone.0080855

**Published:** 2013-12-06

**Authors:** Hua Yang, Zhaojun Li, Lu Lu, Jian Long, Yongchao Liang

**Affiliations:** 1 Institute of Agricultural Resources and Regional Planning, Chinese Academy of Agricultural Sciences, Key Laboratory of Plant Nutrition and Fertilizer, Ministry of Agriculture, Beijing, China; 2 Guizhou Key Laboratory of Mountain Environment, Guizhou Normal University, Guiyang, China; 3 Graduate School of the Chinese Academy of Agricultural Sciences, Beijing, China; University of Kentucky, United States of America

## Abstract

Cadmium (Cd) is a highly toxic heavy metal for both plants and animals. The presence of Cd in agricultural soils is of great concern regarding its transfer in the soil-plant system. This study investigated the transfer of Cd (exogenous salts) from a wide range of Chinese soils to corn grain (Zhengdan 958). Through multiple stepwise regressions, prediction models were developed, with the combination of Cd bioconcentration factor (BCF) of Zhengdan 958 and soil pH, organic matter (OM) content, and cation exchange capacity (CEC). Moreover, these prediction models from Zhengdan 958 were applied to other non-model corn species through cross-species extrapolation approach. The results showed that the pH of the soil was the most important factor that controlled Cd uptake and lower pH was more favorable for Cd bioaccumulation in corn grain. There was no significant difference among three prediction models in the different Cd levels. When the prediction models were applied to other non-model corn species, the ratio ranges between the predicted BCF values and the measured BCF values were within an interval of 2 folds and close to the solid line of 1∶1 relationship. Furthermore, these prediction models also reduced the measured BCF intra-species variability for all non-model corn species. Therefore, the prediction models established in this study can be applied to other non-model corn species and be useful for predicting the Cd bioconcentration in corn grain and assessing the ecological risk of Cd in different soils.

## Introduction

Trace metal elements accumulation in crop of agricultural soils is of extensive concern due to its potential risks to human health and detrimental effects on soil ecosystems [1]–[4]. Cadmium (Cd) is one of the most dangerous trace elements because excessive dietary intake of Cd and its accumulation in human organs over a lifetime can lead to kidney malfunction [5]–[8].

Numerous studies have demonstrated that a number of factors affect Cd bioavailability in soils, including soil pH, organic matter (OM), cation exchange capacity (CEC), cultivars of crop plants, plant age, and so on [9]–[13]. Among these soil properties, the pH plays the most important role in determining metal speciation, due to its strong effects on solubility and speciation of metals in the soil and solution [14]–[15]. Dramatic increases in Cd desorption from soil constituents and its dissolution ability in solution have been observed with the decreasing soil pH [16]–[17]. The bioavailability of Cd in soil also increases when soil pH decreases [18]–[23]. Apart from soil pH, OM in soil is another important factor affecting heavy metal availability. It was reported that heavy metal adsorption onto soil constituents declined when organic matter content in soils decreased [24]–[25], and that the ability of plants to Cd uptake decreased with an increase in soil organic matter [26]–[27].

The influence of soil factors derived from the soil types on plant uptake of heavy metals and the difficulties in assessing the ecological risk of heavy metals in complex soil types have urged the research on prediction models that can predict trace element transfer to plant [28]–[30]. These models provide great opportunities to carry out ecological risk assessments and establish soil quality criteria for heavy metals [31]–[32]. However, the influences of soil factors may result in variability of ecological risk assessments in different soils and of the soil quality criteria for heavy metals [33]. Therefore, when the soil environmental quality standard is established based on the species sensitivity distributions (SSD), which are routinely used in ecological risk assessment procedures [34]–[35], the toxicology data from the soils with different properties need to be normalized using toxicity prediction models to eliminate the influence of soil factors [36]. The normalization of toxicology data can also improve the accuracy of the sensitivity distribution of species and environmental quality standard values [37]. Ideally, separate species-specific models should be developed for each single species. However, this is unrealistic due to the expenses and efforts involved. Thus, for practical reason, the cross-species extrapolation of biological toxicity model was applied in some cases of pollutants risk assessment. For example, Deleebeeck et al. [38]–[39] have applied biotic bigand model (BLM) from O. *mykiss* to other fish species and from D. *magna* to other cladocera species. Schlekat et al. [33] applied chronic nickel BLMs developed for the cladocera such as *Daphnia magna* and *Ceriodaphnia dubia* to predict chronic toxicity of nickel to three other invertebrates including snail (*Lymnaea stagnalis*), insect (*Chironomus tentans*), and rotifer (*Brachionus calyciflorus*). However, little effort has been devoted to establishing models to describe the relationship between Cd uptake of corn grain and soil properties. In addition, the cross-species extrapolation approach has rarely been applied to terrestrial ecosystem. The objectives of this study are: 1) to develop the models for predicting Cd accumulation in corn grain on seventeen soils with greatly different properties, 2) to assess the feasibility of applying these models to other non-model corn species, and 3) to investigate their accuracy in predicting accumulation of Cd in non-model corn species.

## Materials and Methods

None of these 17 soil samples were collected from national parks or other protected areas. It is confirmed that no specific permissions were required for the soils sampling activities in the 17 locations in China. It is also can be confirmed that the field studies did not involve endangered or protected species. No tested corn species are under first- or second-class state protection, and they are not listed in the Inventory of Rare and Endangered Plants of China (http://zrbhq.forestry.gov.cn/portal/zrbh/s/3053/content-457748.html), or the Key Protected Inventory of Wild Plants of China (http://zrbhq.forestry.gov.cn/uploadfile/zrbh/2010-10/file/2010-10-14-bb296addeaa047798d6b6c476aaa1da9.doc). These corn species were used for only scientific research as permitted by the Ministry of Agriculture of China.

### Soil samples

A set of 17 soils covering a wide range of soil properties was collected from typical locations in China. In each sample location, the soils were collected from the top 20 cm of the soil profile. For analysis of their phys-chemical characteristics, the soils were air dried and sorted to pass a 2-mm sieve. Soil pH was measured in deionized water (soil:solution ratio, 1∶5) [40]. Cation exchange capacity (CEC) was determined by the unbuffered silver-thiourea method [41]. OM was measured by dry combustion [42]. The total phosphorus in soils (TP) was measured by colorimetric method [43]. The total nitrogen in soils was determined by the Kjeldahl's method [44]. The background Cd content in the soils was determined by aqua regia (1∶3 fresh mixture of concentrated HNO_3_ and HCl) digestion [45]. The selected properties of the 17 soil samples were shown in Table 1.

**Table 1:** Selected soil properties of the 17 soils used in the cadmium phytotoxicity test.

Soil NO.	Location*	pH	OM (g.kg^−1^)	CEC (cmol. kg^−1^)	TN (g.kg^−1^)	TP (mg.kg^−1^)	TK (g.kg^−1^)	Background Cd (mg.kg^−1^)
S1	Hunan	4.90	15.52	10.85	1.14	468.5	15.26	0.18
S2	Chongqing	5.74	17.48	21.34	1.00	547.8	22.61	0.18
S3	Liaoning	5.74	25.84	12.19	1.00	726.6	23.94	0.16
S4	Yunnan	5.92	34.26	11.10	2.01	811.2	4.77	0.26
S5	Jiangxi	6.01	11.69	8.70	0.51	518.0	9.96	0.14
S6	Anhui	6.25	20.04	19.08	0.99	347.1	15.41	0.17
S7	Heilongjiang	6.27	35.69	28.59	1.74	481.1	24.7	0.18
S8	Jilin	6.82	32.85	31.11	1.75	349.2	24.58	0.17
S9	Jiangsu	6.93	47.69	26.20	2.44	689.2	21.03	0.15
S10	Shaanxi	7.90	16.49	22.37	1.36	984.0	24.37	0.24
S11	Hebei	7.98	8.57	8.12	0.68	529.9	24.22	0.19
S12	Henan	8.07	17.79	16.01	1.07	746.1	19.86	0.19
S13	Xinjiang	8.12	19.43	25.25	1.32	780.0	25.49	0.16
S14	Shanxi	8.24	23.17	16.80	1.13	950.7	23.70	0.20
S15	Tianjin	8.29	22.02	24.67	1.42	916.3	24.63	0.19
S16	Shandong	8.65	11.84	13.09	0.93	965.6	21.37	0.18
S17	Neimeng	8.80	16.30	11.61	0.96	376.3	26.40	0.21

* Soil sample locations are listed in the order of increasing pH.

### Experimental design

#### Bioaccumulation factors for Cd by corn with one species from seventeen soils

According to Grade Two Standard for Cd in the Soil Environmental Quality Standards of China (GB15618–1995), the tested levels of Cd in soils were shown in Table 2. To obtain the tested levels, a certain amount of exogenous Cd (3[CdSO_4_].8H_2_O) was added to the 8 kg air-dried soils. All the soils were thoroughly mixed and placed into pots. Then the soils were moistened with deionized water to the 60% of field moisture capacity. Each tested level of Cd was carried out in triplicate. The soils containing Cd at different levels were covered by plastic film with several pores. For Cd aging, all the pots were kept for 3 months at a greenhouse at the temperature of 25±3°C during the daytime and 20±3°C at night with a natural light photoperiod. During the period of aging, water contents of each soil sample were retained by adding deionized water every 3 days. After 3 months' aging, uniformed seeds of corn (*Zea may* L. cv. Zhengdan 958) were sown in each soil containing Cd.

**Table 2:** Soil Environmental Quality Standards of China (GB15618–1995) and the content of exogenous Cd (mg.kg^−1^).

pH	<6.5	6.5∼7.5	>7.5
Grade Two Standard	0.3	0.3	0.6
Low Cd	0.3	0.3	0.6
High Cd	0.6	0.6	1.0

#### Bioaccumulation factors for Cd of eight corn species from three soils

Three soils with typical properties sampled from Jiangxi, Shaanxi, and Shanxi provinces were selected for this experiment. The tested levels of Cd in soils and exogenous Cd addition were the same as those in the experiment of bioaccumulation factors for Cd by corn from different soils. Triplicate was conducted on each tested level of Cd. After three months' aging of soils, the seeds of corn (cv. Jingketian 183, Chuandan 30, Liaodan 565, Tunyu 88, Zhongdan 808 and Nongda 84) were surface sterilized with 1% (v/v) NaOCl, rinsed, and soaked in distilled water for 24 h at 33°C in the dark. Then these seeds were sown in each soil containing Cd. All these pots were placed in the greenhouse at the temperature of 25±3°C during the daytime and 20±3°C at night with a natural light photoperiod.

### Experimental soil sampling and analysis

After three months' aging, soils in the pots of the above two experiments were sampled, air-dried, passed through a <0.20 mm sieve, and then thoroughly mixed. 0.5 g of the soil sample was weighed and added to digestion tubes containing 9 ml HNO_3_ and 3 ml HF. The corn grains were harvested through being oven-dried at 105°C for 30 minutes and kept at 70°C until the weight of the grains was stable. The samples (0.5 g) were digested in 6 ml concentrated HNO_3_ (70% w/v) and 3 ml H_2_O_2_ in a CEM Mars X microwave oven (CEM Mars) at a pressure of 3.1 MPa. The Cd concentrations in the digestion solution from soil and corn grain samples were measured by inductively coupled plasma-mass spectrometry (ICP-MS, Agilent 7500a, Agilent Technologies Co. Ltd., USA).

### Data analysis

The bioconcentration factor (BCF) was calculated as the ratio of the content of Cd in the corn grain to that in the soil (Equation 1) [46]–[47].
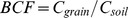
(1)Where *C*_grain_ is the Cd concentration in the corn grain and C_soil_ is Cd concentration in the soil.

SPSS 16.0 for Windows® 107 (SPSS Inc, Chicago, IL, USA) was used for the regression analysis and statistical analysis of the significant differences. Origin 8.0 (OriginLab Co., Northampton, MA, USA) was employed for figure rendering.

### Model

The prediction models for Cd transfer were established through multiple stepwise regression of the Cd BCF from Zhengdan 958 in a wide range of soil properties. These models are based on equation 2.
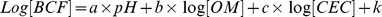
(2)Where Log[BCF], log[OM], and log[CEC] were the logarithm base 10 of the BCF values, the content of organic matter (g.kg^−1^) in soils, and the cation exchange capacity (cmol.kg^−1^) of soils, respectively. The slope of soil property parameters such as a, b and c indicate the impacts of soil properties on heavy metal. The intercept *k* is the intrinsic sensitivity that characterizes the species absorbing Cd.

### Cross-species extrapolation

In the process of cross-species extrapolation, an interim alternative was to assume that interactions among Cd and pH, OM, CEC were the same among related species. In other words, the models stability constants including a, b, c were assumed the same among related species, and the only difference between related species was assumed to be their intrinsic sensitivity (*k*) [33]. The variation of intrinsic sensitivities within a species among plants reflects residual variation [36]. According to the minimum squared error between predicted BCF value and the measured BCF value 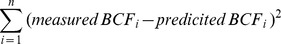, the intercept (*k*) for different models corresponding to various species were obtained through Excel Solver for linear optimization [32].

The accuracy of the model predictions was evaluated by comparing the measured BCF of the other non-model corn species with the predicted BCF from the model. The predictions for other non-model corn species were calculated with the prediction model developed for Zhengdan 958 [48].

### Analysis of the reduction of intra-species variability

The Cd BCF values of non-model corn species were normalized to the specific soil conditions by means of the obtained model for Zhengdan 958 and equation 3.
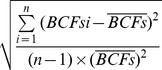
(3)Where the BCF of i-th conditions for specific corn specie was normalized to specific soil conditions. *BCFsi* is the BCF of i-th conditions for specific corn species. 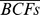 is the mean of n BCFs. *n* is the number of different conditions for specific corn species.

The BCFs that Cd for different corn species was normalized by the prediction models to a set soil condition should be equal. Therefore, the decrease of intra-species variability indicates that normalization processing eliminated soil properties to a certain extent.

## Results

### Major factors affecting accumulation of Cd in corn grain in different soils

**Figure 1:**
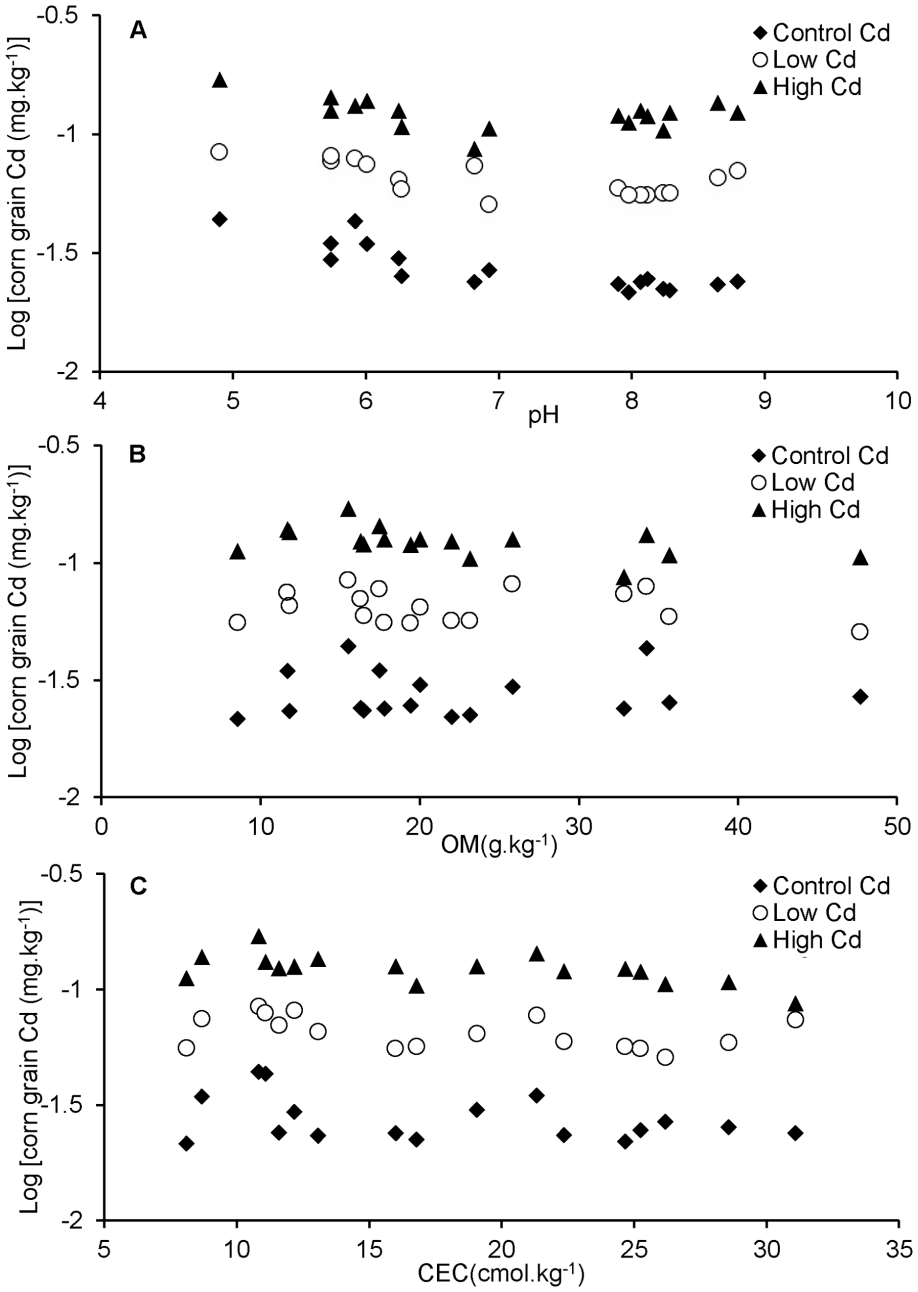
Relationships between Cd in corn grain and (A) soil pH, (B) organic matter (OM) and (C) cation exchange capacity (CEC).

Simple relationships between log [corn grain Cd] at different Cd levels and soil pH, OM and CEC are shown in Figure 1. At the treatment of low concentration Cd, the Cd content in maize grain decreased with an increase of pH. Similar trends were found in the treatments of control and high concentration Cd. The significantly negative correlations were observed between Cd accumulation in corn grain and soil pH in the treatments with control Cd, low concentration Cd, and high concentration Cd (*P*<0.001). There was no significant correlation between the log [corn grain Cd] and soil OM and CEC. These results indicate that soil pH is the main factor that controls the accumulation of Cd in corn grain.

### Effects of different soil types on Cd transfer in soil-corn system

**Figure 2:**
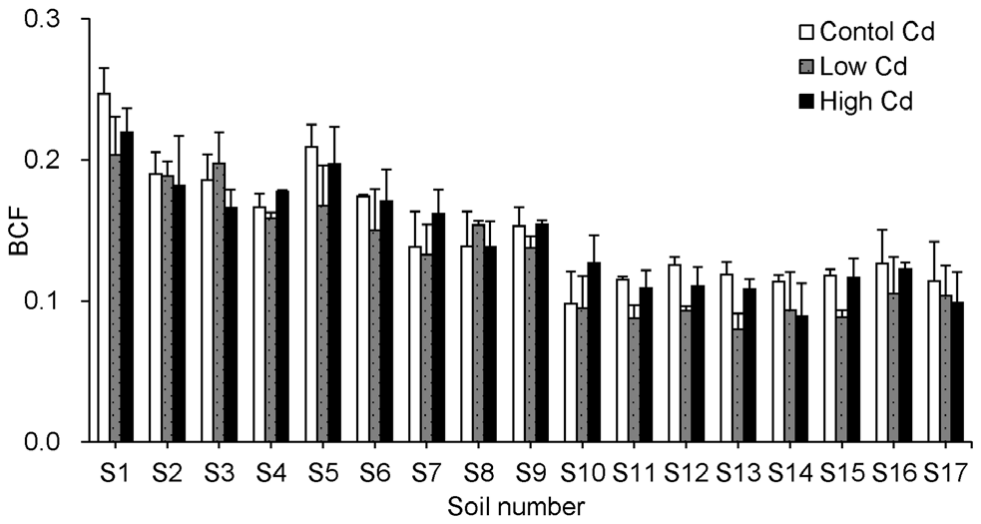
Effects of soil types on the transfer factor based on different Cd treatments in soils.

As shown in Figure 2, the BCF values from cultivar Zhengdan 958 with different treatments were significantly different in 17 soils with a wide range of soil properties in China. In general, the variation tendency of BCF values from cultivar Zhengdan 958 was alike over all soil samples from S1 to S17 at different treatments. These BCF values decreased with the increase of soil pH. The BCF values detected in S1 to S9 (pH from 4.90 to 6.93) were significantly higher than those in S10 to S17 (pH from 7.90 to 8.80). In the control soil, the maximum value for BCF (0.24) was achieved in S1 (pH 4.90), and the minimum value for BCF (0.09) in S10. The maximum value was 2.67 folds as high as the minimum value. At the treatment of low Cd, the maximum BCF (0.20) was observed in S1, and the minimum value for BCF (0.08) was found in S13. The maximum value was 2.50 folds as high as the minimum. At the treatment of high Cd, the highest BCF (0.22) values among the 17 soils were once again obtained in S1, and the minimum value for BCF (0.10) was in S17. The maximum was 2.22 folds as high as the minimum. These results indicate that Cd under acidic soil conditions is more highly bioavailable, leading to increased absorption by plants.

### Prediction models

The prediction models established by stepwise regression are shown in Table 3. A significantly negative correlation existed between the Log[BCF] and soil pH in the Cd treatments including control, low Cd, and high Cd with the R^2^ values ranging from 0.713–0.811 (*P*<0.001). All models displayed the same trend in which Log[BCF] of Cd in corn grain was negatively related to soil pH. There was no significant difference among three prediction models at the different Cd levels.

**Table 3:** Prediction models for the different Cd levels.

Model No.	Cadmium levels	Prediction models	R^2^	*P*
Model 1	Control Cd	Log[BCF] = −0.081pH-0.254	0.728	<0.001
Model 2	Low Cd	Log[BCF] = −0.104pH-0.170	0.811	<0.001
Model 3	High Cd	Log[BCF] = −0.079pH-0.280	0.713	<0.001

### Cross-species extrapolation

The intercept (*k*) for different corn species deduced by three different models are shown in Table 4. The intercept (*k*) means the sensitivity of corn species to Cd accumulation. No significant differences were found to exist either among the *k* values of all tested non-model corn species deduced by the same model (model 1, 2, or 3) or among those in each of the tested non-model corn species deduced by different models (model 1, 2, and 3).

**Table 4:** Intrinsic sensitivity (*k*) for non-model species fitted by models from cultivar Zhengdan 958.

	Intrinsic sensitivities (*k*)					
Prediction Model	Jingketian 183	Chuandan 30	Liaodan 565	Tunyu 88	Zhongdan 808	Nongda 84
Model 1	−0.284	−0.284	−0.339	−0.418	−0.324	−0.317
Model 2	−0.128	−0.123	−0.175	−0.254	−0.164	−0.158
Model 3	−0.302	−0.299	−0.353	−0.432	−0.338	−0.331

**Figure 3:**
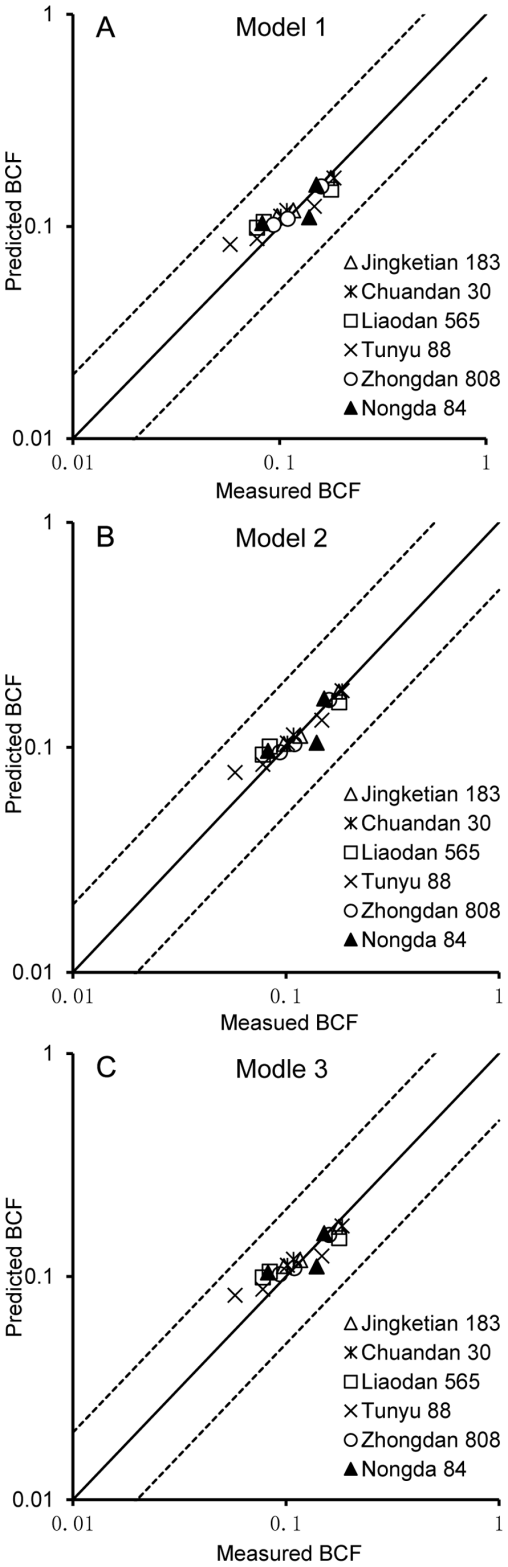
Relativity between measured and predicted BCF values for Cd in non-model corn grains. The predicted BCF values in Figure 3 were estimated by (A) model 1, (B) model 2, and (C) model 3 in Table 3. The solid line represents a 1∶1 relationship; the dashed lines indicate a 2-fold prediction interval between the predicted and measured values.

On the basis of the *k* values, the BCF values of non-model corn species were predicted by different models developed from cultivar Zhengdan958. The relationship between the predicted BCF values and the measured BCF values of non-model corn species was shown in Figure 3. The ratio between the predicted BCF values and the measured BCF values was within 2 folds interval and close to the solid line of 1∶1 relationship, indicating that these models from cultivar Zhengdan 958 can be applied to predict the Cd BCF of non-model corn species.

### Reduction of intra-species variability

**Figure 4:**
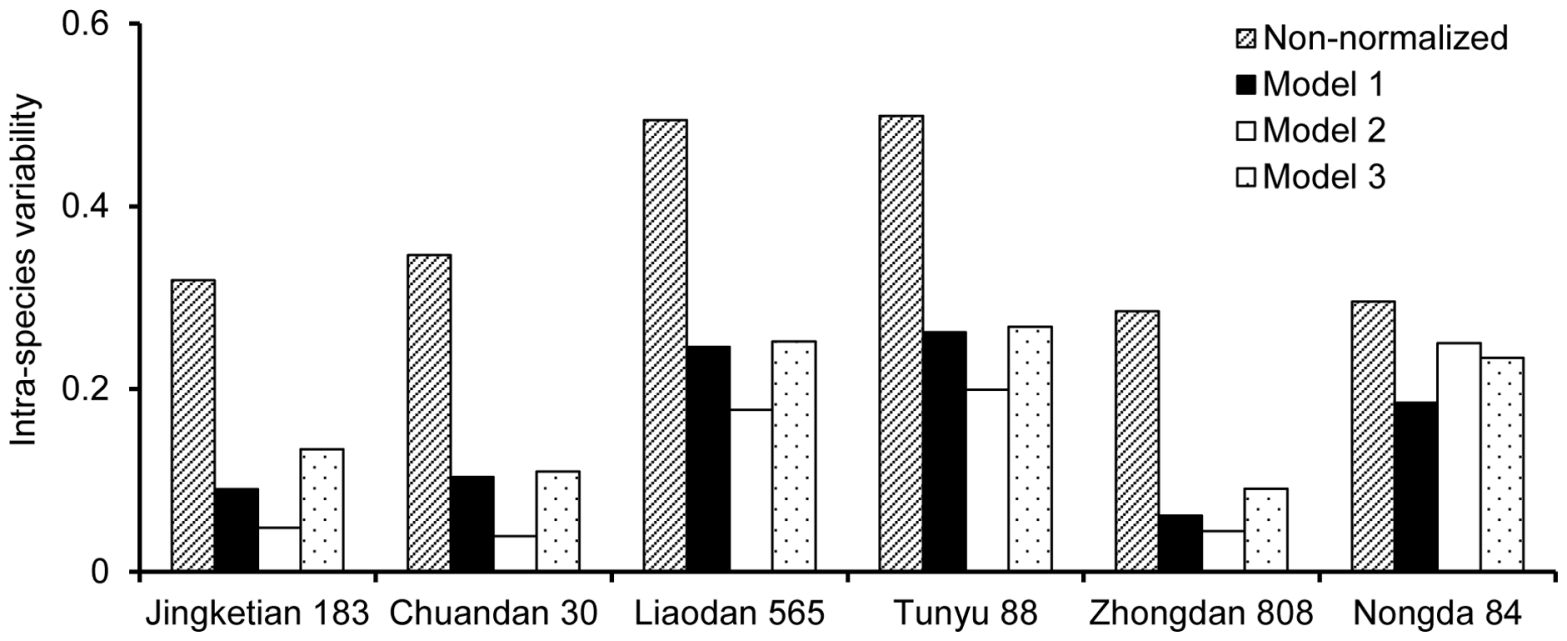
Intra-species variability of Cd BCF The data from all non-model corn species were normalized with the models listed in Table 3.

Figure 4 shows the intra-species variability of Cd BCF of non-model species which were normalized with the models listed in Table 3. The prediction models can reduce the measured BCF intra-species variability for all non-model corn species. The intra-species variability of Cd BCF fitted by model 2 was found much lower than that by model 1 and model 3 except for cultivar Nongda 84. Interestingly, the intra-species variability of Cd BCF for cultivar Nongda 84 normalized with model 1 was much lower than that with model 2 and model 3. No significant differences existed in the intra-species variability of cultivars Chuandan 30, Liaodan 565 and Tunyu 88 which were normalized with model 1 and model 3. Since all models are effective in reducing the uncertainty caused by the soil property differences, they all can be used to establish the soil Cd SSD curves and provide an ecological benchmark in China.

## Discussion

The bioavailable concentration of Cd in soil is more important than its total concentration in terms of its uptake and accumulation in plants [49]. In this study, we found that Cd uptake into corn grain increased with an increase of Cd concentration in soil. However, the BCF values tended to be similar when the concentration of Cd in soil was changed responding to the same soil. These findings were in agreement with previous studies [28]–[30], [50]–[51].

We also found that the soil pH was the major factor influencing the bioavailability of Cd in soil. This may be attributed to Cd uptake by plants from soils through diverse reactions such as absorption, ionic exchange, redox reactions, and precipitation-dissolution etc. [52]. These reactions are mainly affected by soil pH. The pH effects also include its influence on the solution activity of Cd and the distribution of Cd between the soil phase and the solution phase [53]–[54]. The increased sorption of Cd at high pH values could reduce the solution concentration, which tends to decrease the bioavailable concentration of Cd in soil. In this study, the BCF values observed in acidic soils (S1 to S9) were higher than those in alkaline soils (S10 to S17). These results suggest that Cd is much more bioavailable and leads to increased absorption by plants under acidic conditions [55]–[57]. No significant differences were observed among three prediction models at the different Cd levels. There were no significant differences among Cd BCF values of corn grain at different Cd levels, either. These results were consistent with the findings of Rezvani et al. [50].

More importantly, we confirmed that the Cd BCF prediction models from cultivar Zhengdan 958 could be used to predict Cd BCF of other non-model corn species according to the soil pH values through cross-species extrapolation approach. Although many prediction models were developed to describe the relationship between concentration of heavy metals in plants and soil properties in terrestrial ecosystems, these models were only suitable for single species [31]–[32], [58]–[60]. Only a few studies were carried out to develop the prediction models for one species in aquatic ecosystems and subsequently used to normalize ecotoxicity data for other non-model species [33], [38], [39].

**Figure 5:**
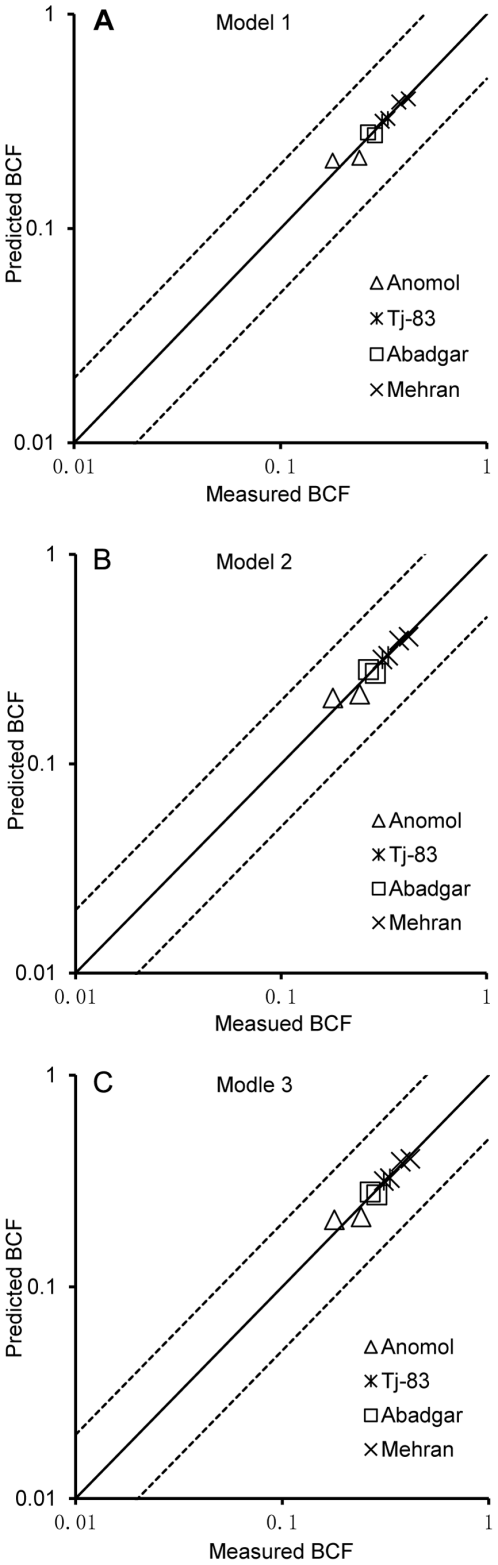
Relativity between measured and predicted BCF values for Cd in non-model wheat grains. The predicted BCF values in Figure 5 were estimated by (A) model 1, (B) model 2, and (C) model 3 in Table 3. The solid line represents a 1∶1 relationship; the dashed lines indicate a 2-fold prediction interval between the predicted and measured values.

**Figure 6:**
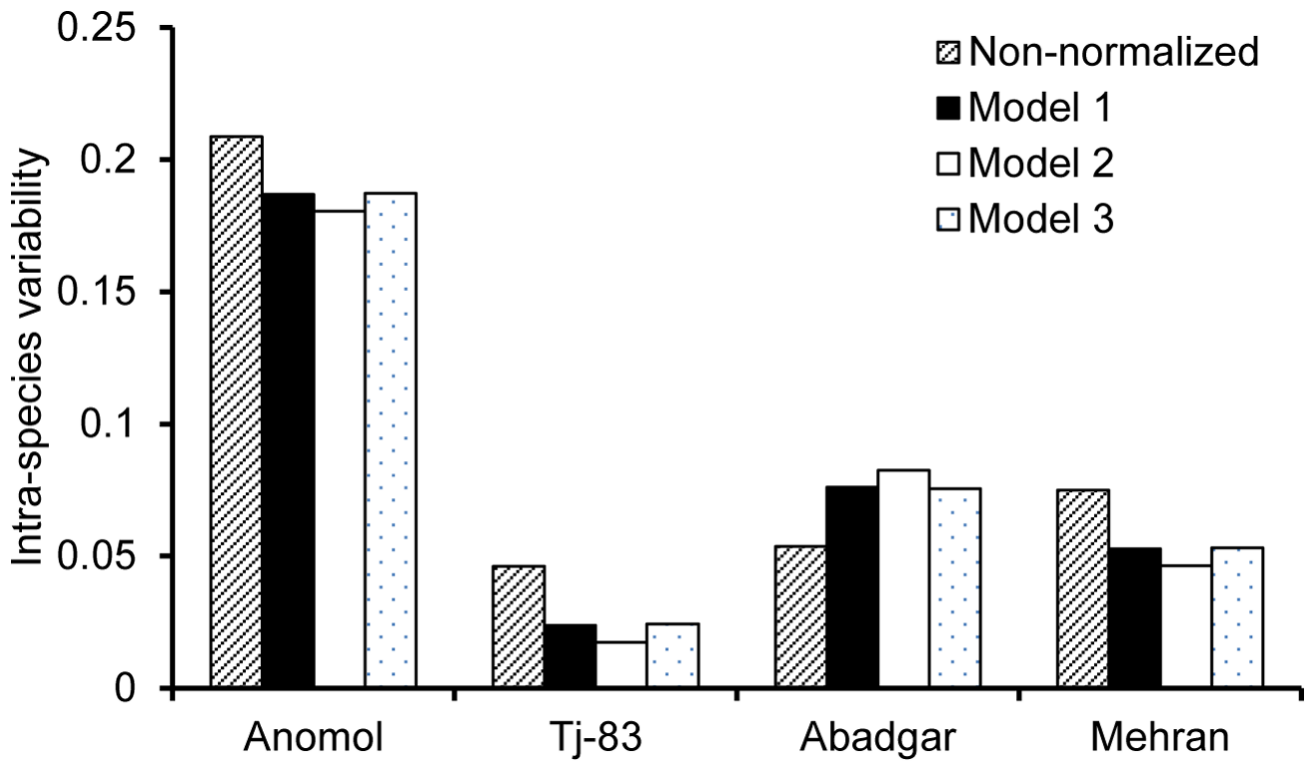
Intra-species variability of BCF for Cd. The data from all non-model wheat species were normalized with the models listed in Table 3.

In order to further validate the feasibility of the prediction modes developed in the present study for other non-model plant species, the measured Cd BCF values of wheat grain from soils were selected from the paper of Jamali et al. [61], and shown in Table 5. Models presented in Table 3 were applied to these non-model wheat species. The relativity between measured and predicted BCF values for Cd in non-model wheat grains is shown in Figure 5. The ratios between all of the predicted BCF values and the measured BCF values were also within 2 folds interval and close to 1∶1 relationship. In addition, the intra-species variability of Cd BCF of all non-model wheat species was decreased except for Abadgar (Figure 6). This indicates that the models from cultivar Zhendan 958 can be applied not only to non-model corn species but also to non-model wheat species.

**Table 5:** Soil properties and BCF values for Cd in non-model wheat grain [61].

				BCF			
Cultivation soil	pH	OM(%)	CEC(mequiv./100g)	Anomol	Tj-83	Abadgar	Mehran
Soil	7.53	75.2	15.0	0.241	0.332	0.266	0.417
Sewage	7.70	87.6	20.8	0.179	0.311	0.287	0.375

## Conclusions

The soil pH was the most important factor that can control Cd uptake and lower pH was more favorable for Cd bioaccumulation in corn grain. The BCF of Cd in corn grain can be predicted by the prediction models such as equation 4. There was no significant difference among three prediction models in the different Cd sources. These prediction models could be applied to other non-model corn species,
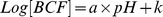
(4)

